# End-of-Life Care Challenges from Staff Viewpoints in Emergency Departments: Systematic Review

**DOI:** 10.3390/healthcare7030083

**Published:** 2019-06-29

**Authors:** Ali J. Alqahtani, Geoffrey Mitchell

**Affiliations:** Primary Care Clinical Unit, Faculty of Medicine, Herston Campus, Royal Brisbane & Women’s Hospital, The University of Queensland, Level 8, Health Sciences Building (16/901), Herston, QLD 4029, Australia

**Keywords:** end-of-life care, palliative care, terminal care, staff view, emergency department

## Abstract

The hospital emergency department (ED) is the place where people most commonly seek urgent care. The initial diagnosis of an end-of-life (EOL) condition may occur in the ED. In this review we described the challenges; from the staff members’ perspectives, to safe, appropriate, and high quality end-of-life care (EOLC) for people who are diagnosed with non-malignant diseases who present to ED settings internationally. We conducted a systematic review of peer-reviewed literature. PubMed, Scopus, CINAHL, Medline, and Web of Science were searched from 2007 to 2017. In this review the challenges in providing quality EOLC from staff viewpoints, for EOL people who are diagnosed with non-malignant progressive diseases in ED settings, were classified into eight themes: (1) EOLC education and training, (2) ED design, (3) Lack of family support, (4) Work Load, (5) ED staff communication and decision making, (6) EOLC quality in ED, (7) resource availability (time, space, appropriate interdisciplinary personnel) and (8) integrating palliative care (PC) in ED. The formulation of EOLC using this review result may help to improve the quality of life for dying people by providing ED staff with clear guidelines that can guide them in their daily practice

## 1. Introduction

The importance of the emergency department (ED) comes from its core mission. EDs play a vital role in providing immediate treatment for patients with acute or traumatic injuries or illnesses [1,2]. The provision of care for patients dying from non-acute conditions is a significant and challenging issue in the ED, a department that has specific characteristics, such as high stress and chaos, that make it different from other departments [2,3,4]. This environment creates an unparalleled combination of challenges that have effects on ED staff practice in making end-of-life care (EOLC) available.

Today, sophisticated medical technologies enable patients to live longer with life-limiting chronic diseases. The ED is often the first step into the healthcare system for patients with poorly managed symptoms of chronic diseases outside the hospital [5], meaning that EDs frequently care for people in need of interventions that include palliative care (PC) and end-of-life care [6,7]. The World Health Organization (WHO) describes palliative care as “an approach that improves the quality of life of patients and their families facing the problem associated with life-threatening illness, through the prevention and relief of suffering by means of early identification and impeccable assessment and treatment of pain and other problems, physical, psychosocial and spiritual” [8]. The term EOLC is usually used following PC to describe the process of care provided to dying patients. However, EOLC has been defined as the care delivered directly prior to death [9]. In this review, the holistic EOLC approach refers to an approach that covers the physical, emotional, and spiritual dimensions of the illness for patients at risk of dying within hours or days.

Guidelines and frameworks in developed countries, such as the United States, the United Kingdom (UK) and Canada, have been formulated to support providing of EOLC in acute settings. The Canadian Nurses Association gives prominence to the implementation of an holistic care provision approach which centralised upon continuity, comfort, palliation and assisting end-of-life (EOL) patients in all clinical settings, including the ED, so that they feel well-supported until death [10]. Furthermore, the guidelines concerning caring for adults who are approaching EOL and who have the probability of dying within 12 months, no matter what their condition or setting is, have been covered by a quality standard released by the UK. Additionally, guidelines related to the provision of EOLC in the last two to three days of life in a clinical setting, such as the ED, have been published by the National Institute for Health and Care Excellence (NICE) in the UK [11]. Having the objective of improving patient outcomes and quality of life, cancer patients have been considered while creating most formulated guidelines and EOLC for particular cancer types, such as advanced breast cancer, has been covered by the formulation of specific NICE guidelines [11]. However, most people do not die from cancer [12,13]. In addition, to the best of our knowledge, while there are some studies on cancer-related palliative care management in ED settings [14,15], there are no such guidelines specific to EOLC for patients with other diseases in ED, such as heart failure. This situation is worse in developing countries- most have no guidelines at all [16].

Little is known about the challenges that ED staff face in implementing high-quality EOLC. Studying the challenges that impede ED staff from providing optimal EOLC to dying patients will help to identify strategies to improve EOLC in emergency departments. This systematic review aims to explore the challenges, from the staff perspective, to safe, appropriate, high-quality EOLC for people who are diagnosed with non-malignant diseases who present to Emergency Department settings.

## 2. Methods

We conducted a systematic review of peer reviewed literature. Electronic searches focused on the challenges, from staff viewpoints, to safe, appropriate, high-quality EOLC in ED settings to people with end stage non-malignant diseases.

### 2.1. Eligibility Criteria

We sought studies from developing and developed countries that reported the views of ED staff who provide EOLC for patients with non-malignant progressive diseases who are considered to be approaching end of life (EOL). In this review, “emergency staff” means any health provider working in an emergency department, which may include nurses, physicians, respiratory therapists, and others. Non-malignant patients, in this review, means any patients suffering a progressive disease other than cancer, in the terminal care stage. The ED, in both private and general hospitals, will be the setting in this review. The inclusion criteria encompass quantitative and qualitative primary studies published between 2007 and 2017. The nature of qualitative studies means they discuss life experience in depth and in an exploratory way from the perspective of staff that work in the ED. Quantitative primary studies, on the other hand, enable an investigation of large numbers of emergency staff views about the challenges of providing quality EOLC. Studies that were not written in English, were focused on cancer, sudden death or trauma patients, or administrative staff, or involved patients under 18 years old were excluded. Moreover, this review excluded articles not published in peer review journals, for example, conference abstracts and theses.

### 2.2. Search Strategy

The search strategy was developed after consultation with an experienced medical science librarian. This librarian is an expert in searching keywords and medical subject headings (MeSHs). Additionally, academic advisors and previous publications provided keyword sources. The search incorporated both keywords and subject headings in the search strategies facilitates the use of different (MeSH) terms, including (“attitude of health personnel” OR “staff view” OR “staff opinion” OR “staff attitude”) AND (“palliative care” OR “terminal care” OR “end of life”) AND (“emergency service” OR “emergency service” OR “emergency department” OR “emergency room”).

### 2.3. Information Sources

Information sources that were searched were the electronic databases PubMed, Scopus, CINAHL, Medline, and Web of Science. A full description of the search terms and strategy is provided in Table S1. Each database has its specific strategy and is available upon request. Retrieved articles were reviewed, particularly their reference lists, which could provide potential studies.

### 2.4. Study Selection

Studies were downloaded and saved online and were managed through Endnote (version X8) (Clarivate Analytics, Philadelphia, PA, USA). The principal reviewer (A.J.A) carried out the initial screening of the abstracts and titles. Then, assessment of full text articles was carried out by two reviewers (A.J.A, G.M.) for inclusion. Lack of agreement was resolved by discussion. Table 1 presents details of the studies and how they were synthesised narratively.

### 2.5. Bias Rating and Quality Assessment

Two reviewers (A.J.A and GM) independently appraised each study for risk of bias and quality assessment using the Effective Public Health Practice Project: Quality Assessment Tool for Quantitative Studies (Table S2). The Standard Quality Assessment Criteria for Evaluation of Primary Research tool was used to evaluate qualitative studies (Table S3).

### 2.6. Synthesis

To synthesise the different designs and methods of the included studies, we used narrative approaches, following the recommended methods developed by Snilstveit et al. [17] particularly content analysis and tabulation. In this review, the process of content analysis was conducted by organising data into categories and elements. The themes were generated by the reviewers (A.J.A and G.M.). Disagreements were resolved by discussion. These themes are presented in Table S4.

## 3. Results

### 3.1. Study Selection

**Figure 1:**
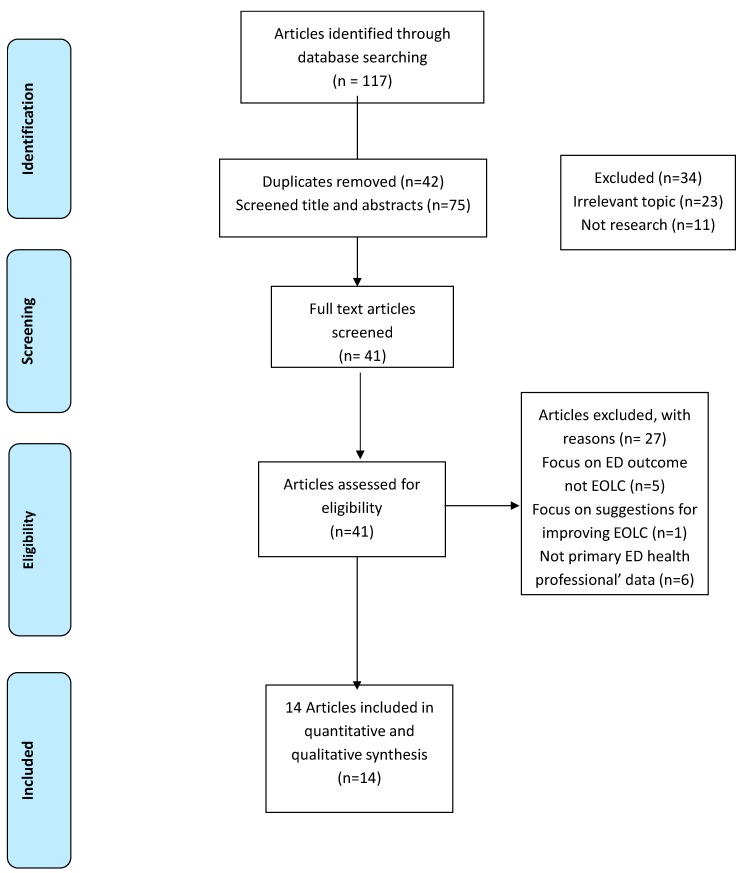
Literature search: screening, inclusion and exclusion.

A total of 117 articles was retrieved, and 14 studies were assessed as meeting the inclusion criteria (see Figure 1). Thirteen studies were conducted in developed countries [3,18,19,20,21,22,23,24,25,26,27,28] and one study was undertaken in a developing country (Thailand) [29]. The quality level of six of the 14 studies was weak [19,20,21,28,30], two studies were of moderate quality [18,24], and six studies were classified as being of high quality [22,23,25,26,27,29]. Five studies of the high-quality studies were undertaken in developed countries, and one study was undertaken in a developing country (Thailand).

### 3.2. Study Characteristics

Eight of the 14 studies were qualitative [18,22,23,24,25,27,29], while five studies used cross-sectional quantitative designs [3,19,20,21,30]. Only one study was based on a mixed-method design [28]. Of the 14 studies, 13 were carried out in public general hospital EDs [3,18,19,20,21,22,23,25,26,27,28,29,30], whereas only one study was conducted in private hospital ED [24]. The sample characteristics of eight studies included only nurses [3,18,19,20,21,22,28,29], while three studies included physicians and nurses [24,25,27]. Three studies incorporated multiple professionals, including physicians and technicians in an ED setting [23,26,30]. See Table 1.

### 3.3. Results

In this review, the challenges in providing quality EOLC, from staff viewpoints, for EOL people who are diagnosed with non-malignant progressive diseases in ED settings, were classified into eight themes: (1) EOLC education and training, (2) ED design, (3) lack of family support, (4) work load, (5) ED staff communication and decision making, (6) EOLC quality in ED, (7) resource availability (time, space, appropriate interdisciplinary personnel) and (8) integrating PC in ED. The full detail is presented in Table S4.

### 3.4. EOLC Education and Training

Nine studies emphasised the importance of EOLC education and training for ED staff in order to provide EOLC in EDs [18,20,23,24,25,26,27,28,30]. A number of studies suggested that insufficient education in regard to EOLC is considered a major barrier to providing quality EOLC for patients [24,26,27,28,30]. Wolf et al. (2015) [28] posited that EOL care should be integrated into nursing education, and they identified the need for clinical practice guidelines to improve EOL nursing skills and patient care. This study was conducted using focus groups with ED nurses who had a specific curriculum focus on nursing knowledge and skills. The authors of this study suggested that nurses’ level of exposure to education sessions related to EOLC might play a role in increasing nurses’ awareness of the importance of EOLC in ED settings. Another study, conducted by Beckstrand et al., (2017) [20] found that the absence of the ideal that nurses considered a “good death” is identified as the most significant obstacle to providing EOLC. Furthermore, Bailey et al. (2011) [27] suggest that the insufficient knowledge and training for ED staff is because ED staff members are trained to provide resuscitation, but not necessarily the palliative care that is needed by most EOL patients. However, Granero-Molina et al. (2016) [25] reported that ED medical staff, including physicians and nurses, warned of their training deficiencies, which leads them to avoid issues related to dying, as they felt unable to deal with the emotional outpourings that usually come with death. A number of studies also reported that most ED nurses noted and accepted the opportunity to be more educated and trained to provide EOLC, as they believe that could fill the service gap in ED [20,24,26].

### 3.5. ED Design

Five studies identified that ED design is a concern for practicing EOLC [3,19,21,22,29], particularly that ED physical design impeded patient privacy. Appropriate ED physical design was considered critical to EOLC in three studies [3,19,22]. These studies suggested that designs of ED are considered as an obstacle to provide EOLC to dying patients [3,19,22]. The respondents felt that EOLC requires a quiet, calm atmosphere to provide patients with quality EOLC and allows them to spend time with their families. This is not the case in ED, as it involves noise and distracting activities. In addition, Hogan et al. [22] determine that the manipulation of physical design in ED, such as dimming the lights, may help to offset these distractions. These studies suggested that patient privacy could not be maintained due to ED design. Kongsuwan et al.’s study [29] found similar issues in Thailand and argued that there is a lack of privacy for a peaceful death. In contrast, Beckstrand et al. (2008) [21] suggested that respondents did not find physical design as a challenge to EOLC were probably working in recently remodelled or new departments. They also felt that nurses who reported a helpful design could be working in departments that were recently built or remodelled.

### 3.6. Lack of Support for Families

Four studies highlighted the lack of support for family members of a dying patient in EDs with three subthemes [3,21,22,25]: the understanding of family members; answering family members’ calls; and support of bereaved family members. Three studies examined the understanding of family members of what was happening to their relative in ED [3,21,25]. Beckstrand et al. (2008) [21] suggested that families expect life-saving interventions to be performed, despite their futility. Nurses face the difficulty of dealing with distraught family members who do not understand that some intensive procedures will inflict pain, or that an endotracheal tube will prevent patients from talking. Granero-Molina et al. (2016) reported that family members insisting on taking a dying patient to ED for an unnecessary examination may result in them dying alone in a cold and impersonal environment [25]. Moreover, answering numerous calls from family and friends is considered an obstacle to EOLC [3]. This study was conducted in a rural ED in a small community where people usually know each other. The authors felt that nurses are not adequately prepared to answer the families’ hard questions regarding their loved ones in ED, especially as the patient or family members could be their friends or neighbours. Hogan et al. (2016) showed that ED nurses are in a position to handle certain challenges when caring for sudden incidents, like supporting bereaved family members, but they are not always fully prepared to support families when their patients is actually dying, such as the case for ICU nurses [22].

### 3.7. Workload

Workload is a theme that was addressed in three studies [20,21,29]. These studies examined the workload of nurses. Beckstrand et al. (2008) [21] and Kongsuwan et al. (2016) [29] identified that the high workload on ED nurses does not permit sufficient time to apply good quality EOLC. Beckstrand et al. (2008) [21] provided further clarification—that the high workload on ED nurses reduces the time available to support patients and their families and provide them with EOLC, which will affect the quality of care that is provided to the EOL patients. A second study Beckstrand et al. (2017) [20] argued that due to the lack of ED staff providing EOLC to terminal patients, nurses’ confidence is negatively affected and the satisfaction with the care provided is diminished.

### 3.8. ED Staff Communication and Decision-Making

This theme is raised in two studies [20,23] as a barrier to making EOLC available in EDs. Beckstrand et al. (2017) [20] found that emergency physicians and nurses often have conflicts about the authority to make decisions. Disagreements between healthcare team members were common occurrences in rural EDs, particularly between physicians and nurses. Nurses commonly related accounts of how such a power struggle impacted the communication process; therefore, nurses felt unsatisfied with the quality of care and believed that their opinion was not valued. The authors concluded that power struggles between physicians and nurses can lead to inappropriate EOLC decisions for terminal patients. In addition, a study conducted in France by Fassier et al. (2016) [23] reported that physicians’ communication was sometimes inadequate and conflicts arose in EDs as a result. They observed that differences in inter-physician communication patterns could influence triage and decision-making. EOLC decisions sometimes occurred as the result of communication between two physicians, while other decisions were carried out by one physician. Both patterns could affect patient safety by coming to inappropriate decisions that might deny or inhibit palliative care. Fassier et al. (2016) [23] noted that EOL decision-making was identified as a challenge to providing appropriate EOLC in EDs, especially when family members are not available or if there is a conflict with family members [23]. By contrast, the authors of this study felt that unilateral decision-making by one physician who does not communicate with other physicians could sometimes be effective when emergency palliative care is needed for EOL patients. Another conflict that has been noted by Fassier et al. (2016) [23] is the one between ED physicians and ICU physicians, which can also affect EOLC. The authors suggested that this conflict may be due to the competition between the physician groups to exercise power over decisions that are made.

### 3.9. EOLC Quality in EDs

Providing quality EOLC in EDs was raised as a challenge in two studies, and this theme includes two different subthemes—improving patient dignity and measuring EOLC [22,25]. First, Granero-Molina et al. (2016) [25] conducted a study in Spain and confirmed that improving dignity for dying patients in EDs is a challenge to providing EOLC. The authors of this study reported that dignity could reflect many attributes, including patients’ preferences, values, lifestyle, respect, autonomy, empowerment and feeling comfortable with oneself. Additionally, this study suggested that the loss of dignity in the ED can be attributed to the lack of standard protocols focused on EOLC and the fact that EDs are designed to save lives, not to provide EOLC. Second, Hogan et al. (2016) [22] reported that measuring quality of EOLC is critical, as it allows for confirmation that the best has been done for dying patients in EDs.

### 3.10. Resource Availability (Time, Space, Appropriate Interdisciplinary Personnel)

Two studies mentioned that restricted availability of resources, including time, space and the lack of an appropriate mix of skilled personnel, is considered an obstacle to providing quality EOLC in ED [18,28]. Wolf et al. (2015) [28] reported that the chaos and noise of the ED is often caused mainly by lack of resources that are usually available in other hospital departments. The authors of this study also suggest that limited time, space and resources affected the nurses’ abilities to provide the EOLC required; as a result, that can increase the feelings of frustration among nurses. Another study found that specific resource allocation for ED EOL services is required [18]. The authors felt that the restriction of nursing time for EOL patients can negatively affect the quality of EOLC, and expressed concerns about EOLC resources that could be used to meet the increasing demand for EOLC, particularly in terms of psychological and spiritual support.

### 3.11. Integrating PC in ED

One of the themes identified is a lack of integration of specialist palliative care teams into EDs. This prevents ED physicians from gaining experience of working through actual cases of terminally ill patients in collaboration with the palliative care team to provide EOLC. In a study conducted in 2011 by Stone et al. [26], it was reported that PC is an important aspect of care for terminal patients in ED. The authors felt that palliative care is not prioritised appropriately, leading patients to be unaware of their choices for EOLC. The same study added that, while ED physicians are willing to utilise the specialist palliative care team, the lack of availability of a SPC team overnight and on weekends remains a concern.

## 4. Discussion

The purpose of this review was to explore the challenges, from the staff perspective, to provide safe, appropriate, high-quality EOLC for people who are diagnosed with non-malignant diseases and who present to Emergency Department settings. It is noteworthy that there was agreement about the identified themes across ED staff. We identified a wide range of challenges of EOLC in EDs for this population. Our findings suggest that the provision of adequate EOLC education and training is considered the most critical challenge to provide EOLC in EDs. Most of the included studies that focused on this point were from ED staff in developed countries. It is difficult to compare these perspectives with those from developing countries due to a lack of studies in these countries. Intensive care clinicians also consider that providing EOLC training and education is essential to providing optimal EOLC for dying patients [31]. This may contribute to the fact that EDs and ICUs share the type of care provided, namely acute and episodic care.

A further crucial challenge that ED staff in most studies greatly stressed with respect to providing good EOLC is ED design. Despite the availability of guidelines for ED design, ED staff surprisingly still consider ED design as an obstacle to provide care for dying patients. By contrast, specific department design has not been considered as a barrier to providing EOLC in other hospital settings, including palliative care and oncology departments. This may reflect the nature of the emergency department, which often generates a high level of noise which can negatively impact the provision of care. In addition, one of the ED staff’s concerns is patient privacy, which is difficult to maintain and will therefore inhibit the ability to provide effective EOLC. A recent review suggested that providing care to advanced cancer patients in ED is also complicated due to its physical design [32]. However, this may suggest that ED staff consider the design of ED as a challenge, without considering the type of life-threating illnesses. It is worth noting that departmental design was not considered as a barrier for other medical staff in any other medical settings.

Furthermore, ED staff identified lack of support for the families of dying patients as a concern. They felt that they are usually under pressure to communicate with families regarding patient care. Although various communication strategies for handling difficult conversations with families have been formulated [33,34], many health professionals try to avoid having EOL conversations with patients when they are discussing a patient’s poor prognosis in the EOL phase [34]. This could be related to many challenges in this situation, as suggested in a recent review [35]. One of these is that patients might be communicatively impaired as a result of a medical condition, such as dementia or a stroke, so symptoms and quality of life cannot be assessed. Another significant challenge affecting EOLC communication is the emotional impact of the EOL discussion on health professionals, as fears and personal experience can prevent them from engaging in EOL discussions. Another major challenge is related to health professionals’ knowledge about when to make the decision to initiate an EOL conversation, as they could be unsure of whether the patient is in the EOL phase. This makes the EOLC process, including supporting the patient’s family, challenging for health professionals.

Another challenge that has been highlighted in this review is staff communication in relation to decision-making. This challenge is quite complicated, as it takes several forms, such as communication between the entire ED staff, which is influenced by different job classifications and a hierarchy of decision-making power. Another form of communication challenge exists between ED staff and medical staff from other hospital departments, such as intensive care unit staff. Disagreements between ED medical staff when making EOLC decisions related to terminal patients might lead to inappropriate quality of care. This might also result in dissatisfaction within some ED staff, such as nurses, as patients might feel and believe their opinions are not valued. Therefore, inappropriate decisions can be made that might affect patient safety. The confliction between physicians from different departments could be due to competition between physicians to exercise power over decisions that are made. This can also influence EOLC decisions negatively, consequently affecting EOLC quality and patient safety. Educating ED staff in how to communicate with other medical staff to provide terminal patients with quality EOLC is recommended. The education in palliative and end-of-life care program (EPEC) [36] is an example of a suitable curriculum to achieve this.

In addition, integrating palliative care into usual ED care is considered a challenge by ED staff. However, medical staff in other departments, such as oncology and palliative care services (PCS), are willing and able to provide EOLC for patients who are at the EOL. Such specialists can provide patients with physical, spiritual, psychological, social and financial support, which are the key components of EOLC. Given the complexity of acute care that is provided in the ED setting, providing EOLC in the ED is challenging.

The numerous challenges of providing quality EOLC studies were explored and assessed in this review, focussing on studies conducted in ED in general hospitals in developed countries. The literature does not clearly show the perspective of ED staff on EOLC in developing countries or whether that perspective differs from, or is similar to, perspectives from developed countries with respect to managing dying patients in ED. Most ED staff have noted that the quality of EOLC in ED is inconsistent. Dissatisfaction with EOLC in ED is often related to the aim and culture of ED care. Evidence from recent studies has shown that ED staff generally perceive that education and training, as well as ED design, are the most significant challenges that require further focus and improvement [32,37]

Globally, research recommendations have informed the updating and modification of certain EOLC guidelines, such as those in Australia. To the best of our knowledge, however, EOLC is still unknown in some developing countries [38,39]. The formulation of EOLC using this review result may help to improve the quality of life for dying people by providing ED staff with clear guidelines that can guide them in their daily practice.

### 4.1. Strengths and Limitations

The strength of this review lies in the fact that the process followed was systematic. This is the first review that has examined ED staff perspectives of the challenges of providing EOLC in EDs. However, this systematic review also has some limitations. Most of the data came from the nursing perspective, so there are little data related to other ED medical and allied health staff. That makes any differences between these professionals much more difficult to identify. Nurses’ data were offered in 11 studies, but other ED medical staff data were found in only six articles. Most of the included studies were from developed countries; only one study was carried out in a developing country (Thailand). Therefore, comparison between developed and developing countries is hard due to limited data from developing countries.

### 4.2. Recommendation for Future Practice

This systematic review provides information which can guide policymakers to develop EOLC approaches in ED settings. It will also help to improve the quality of ED staff members’ everyday practice.

## 5. Conclusions

The purpose of this review was to explore the challenges, from the staff perspective, to provide safe, appropriate, high-quality EOLC for people who are diagnosed with non-malignant diseases and who present to Emergency Department settings. The numerous challenges of providing quality EOLC studies were explored and assessed in this review, focussing on studies conducted in ED in general hospitals in developed countries. The literature does not clearly show the perspective of ED staff on EOLC in developing countries or whether that perspective differs from, or is similar to, perspectives from developed countries with respect to managing dying patients in ED. The formulation of EOLC using this review result may help to improve the quality of life for dying people by providing ED staff with clear guidelines that can guide them in their daily practice.

## Figures and Tables

**Table 1:** Extraction of data from included studies.

Source, Country and Quality	Aim	Design and Method	Setting	Participants
Author: Beckstrand et al., 2008 Country: USA Weak quality * [21]	To determine a magnitude score for both obstacles and supportive behaviours surrounding EOL care in emergency departments.	Cross-sectional using a validated questionnaire	Emergency department, Multisite	272 emergency nurses.
Author: Beckstrand et al., 2012 Country: USA Weak quality * [19]	To determine the impact of ED design on EOL care as perceived by emergency nurses and to determine how much input emergency nurses have on the design of their emergency department.	Cross-sectional using a developed questionnaire	Emergency department, Multisite	198 emergency nurses.
Author: Beckstrand et al. 2012. Country: USA Weak quality * [3]	To discover the size, frequency, and magnitude of obstacles in providing EOL care in rural emergency departments as perceived by rural emergency nurses.	Cross-sectional survey research design	Emergency department in rural area. Multisite	236 emergency nurses
Author: Wolf et al., 2015. Country: USA Weak quality * [28]	To explore emergency nurses’ perceptions of challenges and facilitators in the care of patients at the EOL.	A mixed-methods design	Emergency department, Multisite	Survey data (N = 1879) Focus group data (N = 17)
Author: Beckstrand et al., 2017 Country: USA Weak quality * [20]	To explore the first-person experiences or stories of rural emergency nurses who have cared for dying patients and the obstacles these nurses encountered while attempting to provide EOL care.	Cross-sectional survey	Emergency department, Multisite	246 Emergency nurses.
Author: Hogan et al., 2016 Country: Canada High quality * [22]	To describe the experience of emergency nurses who provide care for adult patients who die in the emergency department to better understand the factors that facilitate care or challenge nurses as they care for these patients and their grieving families.	Qualitative design (Semi-structured interviews)	Two EDs of a multisite university teaching hospital	11 Emergency nurses.
Author: Granero-Molina et al., 2016 Country: Spain High quality * [25]	To explore and describe the experiences of physicians and nurses with regard to loss of dignity in relation to end-of-life care in the emergency department.	Qualitative design (Phenomenological study)	Two EDs of public hospitals, multisite	26 emergency staff (10 physicians and 16 nurses)
Author: Tse, Hung and Pang, 2016 Country: Hong Kong Weak quality * [18]	To understand emergency nurses’ perceptions regarding the provision of EOLC in the ED.	Qualitative study. (Semi-structured, face-to-face interviews)	Emergency Department	16 Emergency Nurses.
Author: Bailey, Murphy and Porock, 2011 Country: UK High quality * [27]	To explore end-of-life care in the ED and provide an understanding of how care is delivered to the dying, deceased and bereaved in the emergency setting.	Qualitative study (Observation and interviews)	ED in an urban academic teaching hospital	Emergency nurses (11), physicians (2), and technicians (2) (7) Patients who had been diagnosed with a terminal illness. (7) relatives, who had accompanied the patients during the emergency admission
Author: Fassier, Valour, Colin and Danet, 2016 Country: France High quality * [23]	To explored physicians’ perceptions of and attitudes toward triage and end-of-life decisions for elderly critically ill patients at the emergency department –ICU interface	Qualitative study (semi-structured interviews)	EDs in Hospitals, multisite	15 Emergency physicians
Author: Stone et al., 2011 Country: USA High quality * [26]	To describe emergency physicians’ perspectives on the challenges and benefits to providing palliative care in an academic, urban, public hospital in Los Angeles	Qualitative study (semi- structured interviews)	ED in a large, public, urban academic medical centre	38 Emergency Medicine Physicians
Author: Kongsuwan et al., 2016 Country: Thailand High quality * [29]	To describe the meaning of nurses’ lived experience of caring for critical and dying patients in the emergency rooms.	Qualitative Study using phenomenological approach (in-depth interviews)	EDs in hospitals, multisite	12 emergency nurses.
Author: Richardson et al., 2016 Country: Australia High quality * [30]	To investigate and describe any differences in the importance of the considerations and discussions that took place when EP and ER made a decision to withdraw and/or withhold life-sustaining healthcare in the ED.	Sub-study of a prospective cross-sectional questionnaire-based case series	In six metropolitan EDs, multisite	185 Emergency consultants, 135 emergency training registrars and 320 EOL patients
Author: Shearer, Rogers, Monterosso, Ross-Adjie and Rogers, 2014 Country: Australia Weak quality * [24]	To investigate Australian ED staff perspectives and needs regarding palliative care provision and to assess staff views about death and dying, and their awareness of common causes of death in Australia, particularly those where a palliative care approach is appropriate.	Qualitative and quantitative survey (The survey tool uses a combination of Likert-type scales and open-ended questions)	In a private ED	22 physicians and 44 nurses

Legend: EOL; end of life, EOLC: end of life care; ED: emergency department; RN: registered nurse; ER: emergency registrar; EP: emergency physician. * Please see Tables S2 and S3 for quality assessment.

## Supplementary Materials

The following are available online at https://www.mdpi.com/2227-9032/7/3/83/s1, Table S1: Terms: PubMed, Table S2: Quality Assessment Appraisal for Quantitative Studies, Table S3: Quality Assessment Appraisal for Qualitative Studies, Table S4: Themes and Sub-themes.
