# Supplementary material for: End-of-Life Care Challenges from Staff Viewpoints in Emergency Departments: Systematic Review
Source: Healthcare (Basel). 2019 Jun 29;7(3):83. doi: 10.3390/healthcare7030083 (PMC6787591; doi:10.3390/healthcare7030083)
Supplement: Supplementary file 1 [file healthcare-07-00083-s001.pdf]

healthcare

*Supplementary Material*

# End-of-Life Care Challenges from Staff Viewpoints in Emergency Departments: Systematic Review

**Ali J. Alqahtani and Geoffrey Mitchell**

**Table S1.** Terms: PubMed.

| | |
|---|---|
| Term 1 | Emergency Service, Hospital, emergency department, emergency room, |
| Term 2 | Terminal Care, Palliative Care, end of life, |
| Term 3 | Attitude of Health Personnel, Health Personnel/psychology, staff views, staff opinion, staff opinions, staff attitudes |
| Complete phrase | ((((“Emergency Service, Hospital”[Mesh]) OR “emergency department” OR “emergency room” OR “emergency rooms”) AND ((“Terminal Care”[Mesh] OR “Palliative Care”[Mesh]) OR “end of life”) AND ((“Attitude of Health Personnel”[Mesh] OR “Health Personnel/psychology” [Mesh]) OR “staff views” OR “staff opinion” OR “staff opinions” OR “staff attitudes”))) |

**Table S2.** Quality Assessment Appraisal for Quantitative Studies.

| Study | Beckstrand et al., 2008 | Beckstrand et al., 2012 | Beckstrand et al., 2012 | Beckstrand et al., 2017 | RICHARDSON et al., 2016 | Wolf et al., 2015 |
|---|---|---|---|---|---|---|
| Selection Bias | 3 | 3 | 3 | 3 | 3 | 2 |
| Study Design | 3 | 3 | 3 | 3 | 3 | 3 |
| Confounders | 3 | 3 | 2 | 3 | 1 | 3 |
| Blinding | 2 | 2 | 1 | 2 | 2 | 3 |
| Data Collection Methods | 1 | 1 | 1 | 1 | 2 | 1 |
| Withdrawals and Drop-out | 1 | N/A | 1 | 3 | 2 | 1 |
| Global Rating | 3 | 3 | 1 | 3 | 3 | 3 |
| Final Decision of both reviewers | WEAK | WEAK | WEAK | WEAK | WEAK | WEAK |

1: strong: 2; Moderate; 3: Weak.

**Table S3.** Quality Assessment Appraisal for Qualitative Studies.

| Study | Bailey et al., 2011 | Fassier et al., 2015 | Granero-Molina et al., 2016 | Hogan et al., 2016 | Kongsuwan et al., 2016 | SHEARER et al., 2014 | Stone, et al., 2011 | Tse, et al., 2016 | Wolf et al., 2015 |
|---|---|---|---|---|---|---|---|---|---|
| Question/objective sufficiently described? | 2 | 2 | 2 | 2 | 2 | 2 | 2 | 2 | 2 |
| Study design evident and appropriate? | 2 | 2 | 2 | 2 | 2 | 1 | 1 | 2 | 2 |
| Context for the study clear? | 2 | 1 | 2 | 2 | 1 | 1 | 2 | 1 | 2 |
| Connection to a theoretical framework/wider body of knowledge? | 2 | 1 | 2 | 0 | 2 | 1 | 1 | 0 | 1 |
| Sampling strategy described, relevant and justified? | 1 | 2 | 2 | 1 | 2 | 1 | 0 | 0 | 2 |
| Data collection methods clearly described and systematic? | 2 | 2 | 2 | 1 | 1 | 1 | 1 | 1 | 2 |
| Data analysis clearly described and systematic? | 2 | 2 | 1 | 2 | 2 | 1 | 2 | 2 | 2 |
| Use of verification procedure(s) to establish credibility? | 2 | 1 | 2 | 2 | 2 | 0 | 1 | 2 | 2 |
| Conclusions supported by the results? | 2 | 2 | 2 | 2 | 2 | 2 | 2 | 1 | 2 |
| Reflexivity of the account? | 2 | 2 | 0 | 2 | 2 | 1 | 1 | 0 | 2 |
| Overall scores out of 20 | 19 | 17 | 17 | 16 | 18 | 11 | 13 | 11 | 19 |

2: Yes; 1: Partial; 0: No.

**Table S4.** Themes and Sub-themes.

**1-EOLC education and training.**

| Sub-Theme / REF | EOLC Training | EOLC Education | Profession Attitude |
|---|---|---|---|
| Author: Wolf et al., 2015. Country: USA | Concerns around challenges with EOL care, appropriate training for nurses. | Dissonance between the nature of emergency care and the nature of EOL care | |
| Author: Beckstrand et al., 2017 Country: USA | The lack of an ideal death (e.g., the nurse personally knows the patient, issues with family members, and unknown patient wishes) was the top obstacle | | |
| Author: Granero-Molina et al., 2016 Country: Spain | "Lack of a palliative culture" | | "Being self-critical with professional attitudes,": Nurses noted that they treat patients in impersonal and dehumanized way and Such attitudes cannot be excused by situations of overcrowding. |
| Author: Tse, Hung and Pang, 2016 Country: Hong Kong | enhancing personal growth and professionalism | | |
| Author: Bailey, Murphy and Porock, 2011 Country: UK | | Dissonance between the nature of emergency care and the nature of EOL care | Care quality dissatisfaction |
| Author: Fassier, Valour, Colin and Danet, 2016 Country: France | | | Physicians revealed a representation of elderly patients that comprised both negative and positive stereotypes, and expressed the concept of physiologic age. These age-related factors influenced physicians' decision making in resuscitate/not resuscitate situations. |
| Author: Stone et al., 2011 Country: USA | | Respondents felt that palliative care is not prioritized appropriately, leading patients to be unaware of their options for end-of-life care. Providing educational materials and courses that have been developed from the ED perspective should be included in ongoing continuing medical education. | |
| Author: Richardson et al., 2016 Country: Australia | Focused training may improve decision-making consistency between emergency physicians (EP) and emergency registrar (ER). | Focused education may improve decision-making consistency between EP and ER. | That there are differences between EP and ER in the level of treatment provided, rates of withdrawal, the considerations that were rated important and the time to death for patients. It also showed that ER made a significant number of end-of life decisions on their own. |
| Author: Shearer, Rogers, Monterosso, Ross-Adjie and Rogers, 2014 Country: Australia | No clinical proficiency in symptom control. | Most reporting only working knowledge of palliative care but clinical proficiency in symptom control. | |

**2-ED design.**

| Sub-Theme / REF | ED Physical Design | Pt Privacy |
|---|---|---|
| Author: Beckstrand et al., 2008<br>Country: USA | Poor design of emergency departments | |
| Author: Beckstrand et al., 2012<br>Country: USA | Nurses did not report that ED design was as large an obstacle to EOL care as previous studies had suggested. Nurses reported that the ED design helped EOL care at a greater rate than it obstructed EOL care | The most common request for design change was private places for family members to grieve |
| Author: Beckstrand et al. 2012.<br>Country: USA | The poor design of emergency departments that does not allow for privacy of dying patients or grieving family members | |
| Author: Hogan et al., 2016<br>Country: Canada | The environment made it difficult to care for dying patients and their families because of unpredictability, busyness, noise | The environment made it difficult to care for dying patients and their families because of lack of privacy |
| Author: Kongsuwan et al., 2016<br>Country: Thailand | | Lacking for privacy for peaceful deaths |

**3-Lack of family support.**

| Sub-Theme / REF | Understanding of Family Members | Answering Family Members' Calls during Their Shifts | Support of Bereaved Family Members |
|---|---|---|---|
| Author: Beckstrand et al., 2008<br>Country: USA | Family members not understanding what "life-saving measures" really mean | | |
| Author: Beckstrand et al. 2012.<br>Country: USA | The top 3 perceived obstacles by rural emergency nurses were: (1) family and friends who continually call the nurse for an | The top 3 perceived obstacles by rural emergency nurses were: (1) family and friends who continually call the nurse for an | |
| Author: Hogan et al., 2016<br>Country: Canada | | | These nurses were also put in the position of caring for the suddenly bereaved family members, which was viewed as an especially challenging aspect of their role |
| Author: Granero-Molina et al., 2016<br>Country: Spain | "Family obstinacy and hospital rescue," with the subthemes "making ill-advised choices" and "avoiding burden." | | |

**4-Work Load.**

| Sub-Theme / REF | Work Load |
|---|---|
| Author: Beckstrand et al., 2008<br>Country: USA | ED nurses' workloads being too high to allow adequate time for patient care |
| Author: Beckstrand et al., 2017<br>Country: USA | Insufficient ED staff |
| Author: Kongsuwan et al., 2016<br>Country: Thailand | "no time for palliative care" |

**5-ED staff communication and decision making.**

| Sub-Theme / REF | Conflicts between Physicians and Nurses | Physicians' Communication | EOL Decision Making |
|---|---|---|---|
| Author: Beckstrand et al., 2017<br>Country: USA | Power struggles between nurses and physicians | | |
| Author: Fassier, Valour, Colin and Danet, 2016<br>Country: France | | Three main communication patterns framed the decisions: interdisciplinary decisions, decisions by 2 physicians on their own, and unilateral decisions by 1 physician.<br>• Conflicts and communication gaps occurred at the ED | End-of-life decisions were perceived as more complex in the ED, in the absence of family or of information about elderly patients' end-of-life preferences, and when there was conflict with relatives, time pressure, and a lack of training in end-of-life decision making.<br>During decision-making, patients' safety and quality of care were potentially compromised by delayed or denied intensive care and lack of palliative care. |

**6-EOLC quality in ED.**

| Sub-Theme / REF | Improving Dying Person Care | Measuring EOLC |
|---|---|---|
| Author: Hogan et al., 2016<br>Country: Canada | | "Needing to know you've done your best." |
| Author: Granero-Molina et al., 2016<br>Country: Spain | "Being exposed in a cold world," with the subtheme "improvising dying person care" | |

**7-Recourses availability (time, space, appropriate interdisciplinary personnel).**

| Sub-Theme / REF | Recourses Availability (Time, Space, Appropriate Interdisciplinary Personnel) |
|---|---|
| Author: Wolf et al., 2015.<br>Country: USA | A lack of resources to provide this type of care in the emergency setting. |
| Author: Tse, Hung and Pang, 2016<br>Country: Hong Kong | Expressing ambiguity toward resource deployment |

**8-Integrating PC in ED.**

| Sub-Theme / REF | Integrating PC in ED |
|---|---|
| Author: Stone et al., 2011<br>Country: USA | Palliative care is very important to provide even in the challenging setting of the ED. In general, respondents spoke of these benefits in three distinct categories: patient, family, and provider benefits.<br>Patients: Pain management &Quality of life<br>Families: More prepared for death<br>Health providers: Training can broaden perspective for emergency physicians in training & Increased job satisfaction |

© 2019 by the authors. Licensee MDPI, Basel, Switzerland. This article is an open access article distributed under the terms and conditions of the Creative Commons Attribution (CC BY) license (http://creativecommons.org/licenses/by/4.0/).
